# Functional Carbon-Based Materials for Blood Purification: Recent Advances Toward Improved Treatment of Renal Failure and Patient Quality of Life

**DOI:** 10.3390/bioengineering12080893

**Published:** 2025-08-21

**Authors:** Abolfazl Mozaffari, Farbod Alimohammadi, Mazeyar Parvinzadeh Gashti

**Affiliations:** 1 Department of Polymer and Textile Engineering, Yazd Branch, Islamic Azad University, Yazd 14778-93855, Iran; 2 Department of Civil and Environmental Engineering, Temple University, Philadelphia, PA 19122, USA; farbod.alimohammadi@temple.edu; 3 Department of Chemistry, Pittsburg State University, 1701 South Broadway Street, Pittsburg, KS 66762, USA; 4 National Institute for Materials Advancement, Pittsburg State University, Pittsburg, KS 66762, USA

**Keywords:** functional carbons, hemoperfusion, hemodialysis, oral adsorbents, blood toxins, renal failure

## Abstract

The accumulation of blood toxins, including urea, uric acid, creatinine, bilirubin, p-cresyl sulfate, and indoxyl sulfate, poses severe health risks for patients with renal failure. Effective removal strategies are essential to mitigate complications associated with chronic kidney disease (CKD) and improve patient outcomes. Functional carbon-based materials, such as activated carbon (activated charcoal) and graphene oxide, have emerged as promising adsorbents due to their large surface area, adjustable porosity, and biocompatibility. This review comprehensively explores the latest advancements in carbon-based materials for blood purification across three key therapeutic modalities: (1) Hemoperfusion, where activated and modified carbonaceous materials enhance the adsorption of small-molecule and protein-bound toxins; (2) Hemodialysis, where functionalized carbon materials improve clearance rates and reduce treatment duration; and (3) Oral Therapeutics, where orally administered carbon adsorbents show potential in lowering systemic toxin levels in CKD patients. Furthermore, we present a comparative analysis of these approaches, highlighting their advantages, limitations, and future research directions for optimizing carbon-based detoxification strategies. The findings discussed in this review emphasize the significance of material engineering in advancing blood purification technologies. By enhancing the efficiency of toxin removal, carbon-based materials have the potential to revolutionize renal failure treatment, offering improved clinical outcomes and enhanced patient quality of life.

## 1. Introduction

Chronic kidney disease (CKD) is a global public health concern characterized by the gradual loss of kidney function, which can progress to end-stage renal disease requiring dialysis or kidney transplantation [1,2]. The accumulation of uremic toxins in the blood—particularly those derived from gut microbiota such as indoxyl sulfate and p-cresyl sulfate—has been strongly linked to both the progression of CKD and the development of cardiovascular complications [3,4,5]. Our previous research has demonstrated that excessive consumption of dietary supplements may exacerbate the risk of kidney stone formation and chronic kidney disease (CKD) [6,7,8,9].

Uremic toxins are generally categorized into four major groups: (i) small, water-soluble molecules (e.g., urea and creatinine); (ii) protein-bound compounds (e.g., indoxyl sulfate, hippuric acid); (iii) larger molecules such as β2-microglobulin; and (iv) various pro-inflammatory cytokines (e.g., IL-6 and IL-8) [10]. The artificial elimination of these toxins is mainly accomplished through blood purification methods, including hemodialysis, hemoperfusion, and, more recently, oral adsorption therapies [11].

Advancements in material science have led to the development of a broad range of adsorbents capable of efficiently removing diverse uremic toxins [12]. Among these, carbon-based adsorbents such as activated carbon (AC, also known as activated charcoal) and graphene oxide (GO) are particularly promising due to their large surface areas, tunable pore structures, and excellent adsorption properties [13,14,15]. The key performance metrics of any adsorbent include adsorption capacity, selectivity, kinetics, biocompatibility, cost-efficiency, and reusability. Adsorption capacity, in particular, is governed by equilibrium dynamics and is a primary factor influencing the efficacy of blood purification processes [12,13].

Porous adsorbents—ranging in pore size from nanometers to micrometers—are often fabricated from both natural and synthetic sources [16]. Common inorganic adsorbents include alumina, silica, zeolite, and novel mesoporous frameworks [12,13,16]. Meanwhile, polymeric materials such as cellulose derivatives, chitosan, and synthetic copolymers can be chemically modified or grafted with functional ligands to tailor their adsorption properties for biomedical applications [12,13,16,17,18].

Activated carbon was among the earliest materials used for extracorporeal blood purification and remains a fundamental component in current treatment methods [13,14]. Recently, interest has grown in next-generation carbon nanomaterials—such as hollow mesoporous carbon spheres, carbon nanotubes (CNTs) and three-dimensional graphene —due to their high surface area, rapid adsorption kinetics, and excellent hemocompatibility [13,14,19,20,21,22,23].

Although commercial adsorbents and resins are available, clinical applications still encounter challenges, such as prolonged treatment durations, high operational costs, and limited efficiency for protein-bound and mid-molecular-weight toxins [24]. In response, previous studies have concentrated on developing functionalized carbon-based adsorbents to enhance the efficacy and selectivity of blood purification systems [23,24].

Given the versatility, cost-effectiveness, and tunability of carbon materials, this review explores the application of two major carbon-based adsorbents—activated carbon and graphene oxide—for blood purification purposes [13,25]. We discuss their physicochemical properties, biocompatibility, adsorption mechanisms, and clinical performance across three therapeutic approaches: oral carbon administration, hemodialysis, and hemoperfusion systems.

## 2. Overview of Blood Purification Methods in Kidney Disease Management

### 2.1. Hemodialysis (HD): Diffusion-Based Toxin Removal with Emerging Carbon Integration

**Figure 1:**
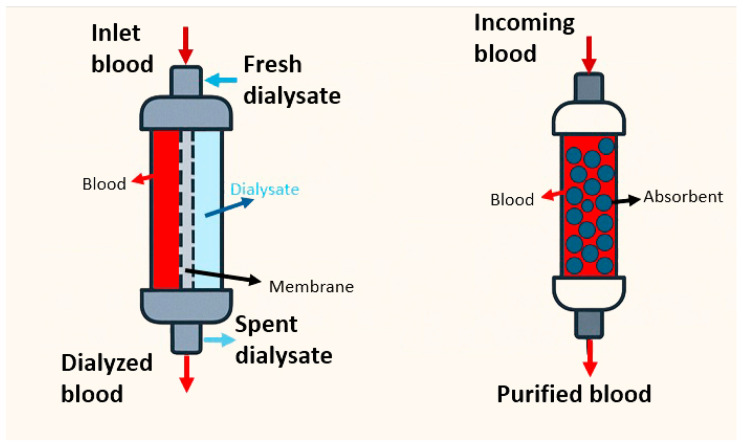
Schematic comparison of hemodialysis and hemoperfusion systems. In hemodialysis (**left**), blood flows through a dialyzer separated from the dialysate by a semi-permeable membrane, enabling diffusion-based toxin removal. In hemoperfusion (**right**), blood passes directly through an adsorbent-packed cartridge, allowing direct contact between blood and carbon-based materials for toxin adsorption.

Dialysis is a widely-established medical procedure that utilizes an external machine to filter waste products and excess fluids from the blood [26,27]. There are two routine types of dialysis, namely hemodialysis and peritoneal dialysis. Hemodialysis involves directing blood to an external machine where it passes through semi-permeable membranes (dialyzer) before being returned to the body [27,28]. Blood passes into the dialyzer and through a semi-permeable membrane, allowing toxins to diffuse into the dialysate on the other side [29]. Figure 1 illustrates two blood purification methods used in renal failure treatment: conventional dialysis, where waste products diffuse across a semi-permeable membrane into a dialysate, and hemoperfusion, where blood passes through an adsorbent-filled column that directly removes toxins. These schematics highlight the fundamental differences in the mechanisms of solute removal between diffusion-based dialysis and adsorption-based hemoperfusion.

The purification process by hemodialysis was first introduced in the 1970s and 1980s, by regenerating and reusing the dialysis solution (dialysate) instead of discarding it [30]. The extracellular components in dialysate fluids may be adjusted or removed by sorbent materials [9]. The most conventional sorbents to remove these toxins have been carbon-based materials [31,32]. Activated carbons (ACs), recognized from ancient times [33], are considered to be included among the most effective adsorbents, mainly owing to their highly enlarged porous structure and the large surface area [12]. Highly purified AC can be made hemocompatible; in practice, blood-contacting HP cartridges often employ polymer coatings (e.g., cellulose derivatives) to improve biocompatibility [34,35,36,37,38,39]. ACs are also the most well-known adsorbents for removing organic molecules in cases of poisoning [33]. Recent studies published by the American Academy of Clinical Toxicology and the European Association of Poisons Centers and Clinical Toxicologists on the medical applications of ACs recommended AC products for treating ingested intoxications [40,41].

The surface modification of carbon-based materials is pivotal for enhancing their performance in filtration applications, especially in hemodialysis and hemoperfusion [42,43,44]. Targeted modifications can markedly enhance the adsorptive properties of these materials, which is critical for the effective removal of contaminants from biological fluids [42,43,44]. Among the various strategies employed, chemical treatments are commonly used to introduce functional groups that improve the interaction between the carbon surface and solutes [42,43,44]. In particular, the UV/H_2_O_2_ advanced oxidation process has demonstrated efficacy in regulating both the functional group composition and pore structure of carbon materials, thereby improving their contaminant removal capabilities [42,43,44]. These modifications are essential, as the chemical characteristics of carbon surfaces strongly influence their affinity for diverse solutes, including endogenous toxins and pharmaceutical residues [45]. Furthermore, adding oxygen-containing functional groups improves hydrophilicity, facilitating interaction with polar compounds typically found in biological fluids [46].

Clinically, activated carbon (AC) plays a crucial role in sorbent-based dialysis. In recirculating dialysate (REDY) systems, spent dialysate flows through an activated-carbon sorbent cartridge that removes uremic solutes; the regenerated dialysate is then returned to the dialyzer [30]. It is worth mentioning that only the dialysate—not the blood—passes through the sorbent bed [30]. AC-based sorbent dialysis targets a broad spectrum of uremic solutes (~264), with removal occurring as the spent dialysate passes through the activated-carbon cartridge [47]. Beyond dialysate regeneration, AC is applied (i) as oral adsorbents (tablets/capsules) to bind toxins in the gastrointestinal tract and (ii) in extracorporeal sorbent devices such as hemoperfusion columns that directly contact blood to remove protein-bound or hydrophobic compounds [33].

### 2.2. Hemoperfusion (HP): Direct Blood Contact with Activated Carbon for Toxin Adsorption

Hemoperfusion is an extracorporeal blood purification technique that utilizes adsorbent materials, primarily activated carbon, to remove toxins from the bloodstream [48]. This method has been particularly effective in treating drug overdoses and certain types of poisoning [48]. In hemoperfusion, blood is drawn from the patient and passed through a column containing activated carbon or other microporous materials [49]. This selective adsorption process is crucial for effectively removing harmful substances from the blood without damaging the formed elements of blood [48,50]. The AC adsorbs toxins directly from the blood, thereby reducing the concentration of toxins [13]. This method can be effective in cases of acute poisoning, as it enables the rapid removal of a broad range of toxins from the bloodstream [49].

Hemoperfusion using activated carbon has shown effectiveness in removing various toxins, including those that are difficult to eliminate via conventional hemodialysis including protein-bound and lipophilic (fat-soluble) toxins [51]. However, it is essential to monitor for potential complications such as thrombocytopenia and other hematological effects due to the nature of the procedure [51]. Despite these risks, hemoperfusion remains a valuable tool in emergency medicine for managing severe poisoning cases [51].

### 2.3. Oral Carbon Adsorbents: Gastrointestinal Toxin Removal Using Carbon-Based Therapies

Recent advances in oral carbon-based therapeutics have introduced promising strategies for managing chronic kidney disease (CKD) by reducing the systemic accumulation of uremic toxins [52,53]. One such approach involves Oral Spherical Carbon Adsorbents (OSCA), which have been developed specifically to target indoxyl sulfate (IS), a protein-bound uremic toxin associated with cardiovascular complications in CKD patients [52,53]. In a clinical study involving patients with moderate to severe CKD, OSCA treatment significantly reduced serum IS levels by 22.5% at 4 weeks and 31.9% at 8 weeks [52,53]. Similarly, oral activated charcoal has been evaluated for its ability to decrease serum levels of urea, creatinine, and phosphorus [52,53]. In a randomized controlled trial, CKD patients administered 3 g of activated charcoal daily showed marked reductions in serum urea and creatinine over a 12-week period compared to the control group [54]. Both OSCA and activated charcoal function primarily through adsorption, binding toxins within the gastrointestinal tract and preventing their systemic absorption [52,53]. This mechanism is especially beneficial for CKD patients who cannot efficiently eliminate these toxins due to impaired renal function [52,53]. The introduction of oral carbon adsorbents represents a non-invasive and effective adjunctive therapy that may delay disease progression, improve biochemical parameters, and enhance patient quality of life [52,53]. As summarized in Table 1, these approaches offer complementary advantages and challenges, underscoring the importance of tailored treatment strategies based on individual patient needs and toxin profiles.

## 3. Current Kidney Replacement Therapies and Carbon-Based Adsorbents

### 3.1. Role of Activated Carbons in Enhancing Dialysis Efficiency

ACs have been studied as adsorbents to enhance the efficiency of KRT for uremic toxin removal. Activated carbon, comprising over 90% carbon along with oxygen and hydrogen functional groups, presents unique adsorption properties [65,66]. Activated carbons consist largely of sp^2^ carbon with surface oxygen- and hydrogen-containing groups (e.g., hydroxyl, carbonyl, carboxyl), which introduce polarity and govern aqueous adsorption via hydrogen bonding and electrostatic/π-π interactions [65,66].

Adsorption kinetics revealed that urea adsorption increases as the temperature decreases significantly [67,68,69,70]. Creatinine adsorption was also favorable at lower temperatures; however, with slower equilibrium attainment compared to urea [67,68]. Uric acid showed consistent adsorption behavior across temperature ranges, with equilibrium amounts exceeding those of urea and creatinine [69,71]. Thermodynamic analysis via the Gibbs equation indicated that the adsorption processes for these toxins are spontaneous and exothermic, as evidenced by negative Gibbs free energy change (ΔG) and the enthalpy change (ΔH) values [67,68]. A strong correlation was observed among urea, creatinine, uric acid, and bilirubin in these processes [67,68].

In 1948, Muirhead and Reid were the first to explore the potential of sorbent materials for dialysis systems [72]. Later, in 1964, Yatzidis introduced the application of ACs in the hemoperfusion method, demonstrating their ability to adsorb a range of uremic toxins, including creatinine, uric acid, phenols, indolic compounds, guanidines, and organic acids [73]. Despite their promise, the clinical adoption of sorbents was hindered by significant adverse effects, such as poor biocompatibility, platelet loss, and hemolysis of blood cells [28,66,68].

Polymer materials used in blood purification have several drawbacks, such as non-selective adsorption, inappropriate pore sizes, low permeability, and potential toxicity [17].

Researchers have proven that AC can adsorb the urea substance from different solutions [74]. The morphology and functional groups of AC in adsorption characteristics is important for analysis [74]. It has been demonstrated that spherical AC has more prominent resistance, mechanical strength, and adsorptive capacity than both powdered and granulated ones, with some advantages, including small fiery debris substance or pressure loss, high fluidity, bulk density, and the capability to be firmly pressed [39]. Notably, spherical ACs have attracted considerable interest in both gas- and liquid-phase applications [39].

### 3.2. Graphene Oxide Nanomaterials for Renal Replacement and Bioartificial Systems

Carbon-based materials, such as AC particles, hollow mesoporous carbon spheres, and three-dimensional porous graphene, have emerged as promising alternatives to overcome these challenges [14,20]. Their microporous and mesoporous structures make them highly efficient super-adsorbents, providing enhanced selectivity and adsorption capacity [14,20,22,23].

Graphene oxide nanosheets (GOs) have demonstrated size-dependent renal clearance mechanisms, which significantly influence their biological interactions and potential nephrotoxicity [75]. Specifically, small-sized GOs (s-GOs) are primarily excreted via glomerular filtration, whereas large-sized GOs (l-GOs) are predominantly cleared through proximal tubular secretion [75]. These distinct clearance pathways are associated with differential renal compartment interactions, wherein s-GOs tend to induce glomerular alterations, while l-GOs are more likely to cause tubular injuries at higher concentrations [75]. These membranes demonstrate superior molecular sieving properties, facilitating the efficient removal of urea and other uremic toxins while effectively retaining essential plasma proteins, such as albumin [76]. Furthermore, their excellent hemocompatibility, characterized by low hemolysis rates and minimal activation of coagulation pathways, is comparable to or surpasses that of conventional dialysis membrane materials, underscoring their potential for integration into wearable and extracorporeal dialysis systems [76]. In addition, composite hollow fiber membranes incorporating graphene oxide have been explored for use in bioartificial kidney (BAK) systems [77]. These membranes exhibit promising biocompatibility and maintain functional interactions with human renal proximal tubular epithelial cells, which is essential for the development of advanced extracorporeal kidney support devices that aim to replicate both filtration and re-absorptive functions of the native kidney [77]. The advantages and limitations of various functional carbons in blood perfusion treatments are summarized in Table 2.

## 4. Synthesis and Processing of Activated Carbon Materials

### 4.1. Carbonaceous Precursors for Activated Carbon Production

Activated carbon refers to porous carbonaceous products [83,84,85,86,87,88], produced from any carbon-containing material by thermal decomposition or pyrolysis with steam or high temperatures processing (700–1000 °C) [89]. Industrial production typically involves continuous processes such as bead formation, impregnation, and sieving, without the need for surface coating [49]. Common bulk precursors used in large-scale AC manufacturing include peat, lignite, coal, wood, and coconut shells [90]. These materials are favored due to their high carbon content and low ash levels.

In recent years, agricultural waste products—such as vine shoots (Vitis vinifera), cassava peels, and olive pits—have emerged as promising low-cost precursors owing to their sustainability and availability [56]. These raw materials are typically washed, dried, pulverized, and subjected to chemical or physical activation [91].

Activated carbons can be categorized based on physical form and application (Table 3). For example, powdered activated carbon (PAC) offers rapid adsorption due to its fine particle size, while polymer-coated carbon (PCC) is engineered for biomedical uses such as hemoperfusion columns due to its enhanced biocompatibility and structural integrity.

Alongside traditional precursors, a growing amount of research investigates the use of carbonaceous material precursors, such as natural polymers, citric acid, and amino acids, to produce carbon dots, graphene derivatives, and nanoporous carbons via chemical oxidation or hydrothermal carbonization.

These nanocarbons are valued for their tunable surface chemistry, aqueous dispersibility, and biocompatibility, making them suitable candidates for injectable therapies or membrane functionalization in advanced blood purification systems.

### 4.2. Carbonization and Activation Techniques

The ACs are extensively porous adsorbents that are classified into various applications, including dye additives, biomedicals, industries, wastewater treatments, water filtrations, and air pollution treatments [93]. Carbonization and activation are the two critical stages in AC treatments, improving pore structures by physical or chemical activation strategies [56,94].

Activation processes (physical or chemical) influence the surface area and bulk structure of the AC which are related to permeability, chemical nature, and its crystalline structure [56]. The arrangement of the crystalline structure of AC generally starts early in the carbonization preparation stage [56].

Physical activation is carried out through two steps: In the first step (carbonization), the carbonaceous precursor is pyrolyzed at relatively low temperatures (300–600 °C for a maximum of three hours) in the absence of air [91]. The carbonization temperature has the most significant influence, followed by warming rate, nitrogen stream rate, and lastly, residence time [56,95]. The second step involves activating the obtained char beneath an oxidizing air (steam or CO_2_) [56]. In the single-step physical activation preparation, the pyrolysis is carried out beneath an oxidizing gas stream such as steam, carbon dioxide, air, or a mixture [96]. Due to the exothermic reactions between carbon and oxygen (present in air), controlling the reaction temperature is critical, and steam and CO2 play a significant role in this process. [56]. Microporous ACs are arranged by physical activation with CO_2_ as the activating agent [56].

### 4.3. Role of Activating Agents in Surface Development

Activating agents are chemical compounds incorporated into the formulation of precursors to produce activated carbon (AC) [92]. These agents are typically mixed with the precursors prior to the carbonization process to enhance porosity and surface area [92]. Potassium hydroxide (KOH), sodium hydroxide (NaOH), zinc chloride (ZnCl_2_), phosphoric acid (H_3_PO_4_), potassium carbonate (K_2_CO_3_), calcium hydroxide (Ca (OH)_2_), and ferric chloride (FeCl_3_) have been studied as chemical activation agents in the synthesis of activated carbon for potential application in blood purification systems such as hemoperfusion [92,97]. Among these, KOH is the most widely used due to its ability to create well-developed pore structures and high surface area [97], although its direct use in blood-contacting applications is prevented due to its caustic nature; instead, only the processed activated carbon is utilized [97]. ZnCl_2_ and H_3_PO_4_ also serve as effective agents, with ZnCl_2_ requiring careful post-treatment to remove residual zinc ions and ensure biocompatibility, and H_3_PO_4_ offering the advantages of high adsorption efficiency and lower toxicity risk once residual acid is eliminated [98,99,100,101]. NaOH provides a cost-effective alternative to KOH with slightly lower efficiency but acceptable performance when properly treated [102,103], K_2_CO_3_ is valued primarily for handling and process-safety advantages, although its clinical adoption remains limited and typically requires additional surface modification and comprehensive biocompatibility validation [97]. In contrast, Ca(OH)_2_ is not commonly used due to its low activation efficiency and lack of clinical relevance, and FeCl_3_ remains experimental because of concerns over cytotoxicity and insufficient biocompatibility data [104,105]. In summary, calcium-based activation is not a common approach; Ca(OH)_2_ demonstrates limited activation effectiveness, and some studies instead use CaCl_2_—both of which are less common than KOH, ZnCl_2_, or H_3_PO_4_ [30,92,97,106]. The hemocompatibility of the final adsorbent depends on thorough removal of residuals and the surface chemistry of the finished carbon, not on the choice of activator itself. In clinical hemoperfusion, the blood-contacting adsorbent is medical-grade, highly purified activated carbon with additional surface modifications or polymer coatings to meet hemocompatibility standards (e.g., ISO 10993-4) [32,92,97,107].

Table 4 presents key information on various activating agents, emphasizing their functions, limitations, and physicochemical characteristics in the context of activated carbon production. The resulting product generally consists of smooth, non-homogeneous granules with an average particle size of 0.5–1.0 mm and a high specific surface area [108]. Phenol-formaldehyde activated carbon (PFAC), for instance, is a coarse-mesh charcoal with a cinder content below 0.05%. PFAC is synthesized through the carbonization of phenol-formaldehyde resin, followed by activation at 950–1000 °C using carbon dioxide, achieving a burn-off rate of approximately 50% [108].

### 4.4. Synthesis and Structural Features of Graphene-Based Materials for Blood Purification Applications

The structure of the graphite crystal was first explored by Bernal in 1924 using X-ray diffraction [110]. It comprises layers of carbon atoms organized in a honeycomb lattice at about 0.142 nm and planes (d-spacing) at 0.3354 nm [56]. The prominent difference between the structure of graphite and carbon particles arises from the amount and mutual orientation of the crystallites [56]. The crystal structure is exceptionally well-ordered in the graphite matrix but is much more limited in ACs [56].

Graphene oxide (GO) was synthesized from graphite fine powder by modified Hummers’ procedure, utilizing KMnO_4_ and NaNO_3_ concentrated H_2_SO_4_ as reported Kaleekkal and colleagues [111]. Advantages of this process include rate of synthesis, simplicity, relatively safe production, and high production yield [112]. The production of GO from graphite by this strategy is limited owing to the dissemination rate of the oxidizing agents, such as Mn_2_O_7_ and MnO_3_^+^, between the graphene layers [112].

In 1958, Hummers and Offeman presented a method for GO and reduced GO synthesis using a two-step preparation method [23,80,82,112]. In the first step, graphite is oxidized to produce GO [104,105,113,114]. Next, the thermal or chemical reduction of GO results in reduction of GO [104,105,113,114]. With respect to the utilization of low-cost crude materials, and simplicity, this process can be potentially used for large-scale production of functionalized graphene nanomaterials [104,105,113,114]. They employed H_2_SO_4_ and NaNO_3_ for the intercalation of graphite, followed by the introduction of KMnO_4_ to facilitate the oxidation of the graphite [114]. Graphite powder (3.0 g) was included in a mixture of H_2_SO_4_ (12 mL), K_2_S_2_O_8_ (2.5 g), and P_2_O_5_ (2.5 g) in a flask [80,114]. At that point, the blend was heated at 80 °C for 4.5 h for pre-oxidation [80,114]. Progressively, the pre-oxidized powder was filtered and washed with deionized water and dried overnight at room temperature. Accordingly, KMnO_4_ and H_2_SO_4_ were gradually added to assist oxidation [80,114]. After acid treatment, the resulting solid was suspended in deionized water and decontaminated through dialysis to remove any remaining acid and metal species [80,114]. Finally, the remaining brown dispersion was centrifuged for 30 min at 4000 RPM to separate aggregates [80,114]. On the other hand, graphene, a single layer of sp2-bonded carbon atoms arranged in a hexagonal lattice, has garnered substantial interest for its remarkable surface area, electronic conductivity, and chemical stability [82,115]. It also has an array of functional groups, such as carboxyl, hydroxyl, and epoxy bonds, that allow for easy chemical modification [82].

Graphene membranes can be less than one nanometer thick, allowing filtration of nanometer-sized waste molecules from blood at rates up to 10 times faster than current polymer membranes [116]. This dramatically increases dialysis efficiency by enabling quicker removal of toxins like urea and other low molecular weight waste products from the blood [116]. Perforated graphene-based membranes have tunable pore sizes comparable to conventional dialysis membranes but with much higher permeability due to their thinness. This allows selective removal of toxic molecules while retaining essential blood components, improving separation precision and reducing collateral loss of beneficial metabolites during hemodialysis [116]. Incorporating graphene oxide (GO) into membrane materials like polyethersulfone (PES) enhances the membranes’ blood compatibility, reducing blood cell damage, hemolysis, and inflammatory responses [117]. These improvements lower complications during dialysis and reduce the need for anticoagulants [118]. Graphene’s exceptional strength (up to 10 times stronger than steel) contributes to more durable membranes capable of withstanding the stresses of dialysis without compromising performance [116]. The smooth surface of graphene membranes decreases blood clot formation on the membrane’s surface, allowing safer and more efficient blood filtration [116].

## 5. Toxins and Solute Clearance

### 5.1. Organic Solutes and Ionic Species

Healthy kidneys play a crucial role in maintaining physiological homeostasis by efficiently removing metabolic byproducts, commonly referred to as endogenous toxins [11,119,120,121]. In individuals with chronic kidney disease (CKD), there is a significant increase in both morbidity and mortality rates [11,119,120,121]. While some endogenous toxins are water-soluble, the majority exhibit a strong binding affinity to albumin, which facilitates their transport through the bloodstream [122]. These toxin–protein complexes are dissociated via physicochemical interactions with cellular membranes [73]. However, nitrogenous compounds such as salts, urea, uric acid, and creatinine may remain in the bloodstream despite the effective excretion of excess fluids in the urine [73,123,124,125]. This impaired clearance leads to the accumulation of microbiota-derived uremic toxins, including indoxyl sulfate (IS) and p-cresyl sulfate (PCS) [126,127]. Elevated concentrations of these toxins contribute to a range of systemic complications, most notably cardiovascular disease and infection, thereby reducing quality of life and impairing daily functioning [71,119,125,128,129,130,131].

In hemodialysis, small, water-soluble solutes (e.g., urea, creatinine, uric acid; <0.5 kDa) can be removed primarily by diffusion into the dialysate. Middle molecules (~0.5–60 kDa) and protein-bound uremic toxins are less efficiently cleared by diffusion and often require high-flux membranes and/or convective or adsorptive strategies. The dialysate is an electrolyte solution (typically Na^+^, Cl^−^, HCO_3_^−^ buffer, Ca^2+^, Mg^2+^, K^+^, ±glucose) formulated to be near-isotonic with plasma [30,107,125,132,133,134,135].

The European Working Group on Uremic Toxins (EUTox) has cataloged more than 90 distinct uremic toxins, which are classified into three principal categories based on their molecular weight and chemical characteristics [123]. This classification system has direct implications for the efficacy of toxin removal and the selection of appropriate extracorporeal purification techniques [73].

Uremic toxins are broadly grouped into the following categories according to their molecular size and protein-binding characteristics:

i. Water-Soluble, Low-Molecular-Weight Compounds (<0.5 kDa)

This group includes small solutes such as urea and creatinine, which are efficiently removed by standard dialysis modalities [136,137,138]. Creatinine, a byproduct of muscle metabolism, is widely recognized as a biomarker of renal function [88]. Elevated serum creatinine concentrations are indicative of reduced kidney clearance capacity and often serve as an early sign of renal impairment prior to the onset of overt clinical symptoms [88]. These substances are commonly referred to as “uremic retention solutes”; when they exhibit biological or chemical activity that contributes to pathological states, they are designated as “uremic toxins” [123]. The accumulation of such compounds contributes to chronic endogenous intoxication and progressive physiological deterioration [73].

ii. Middle-Molecular-Weight Compounds (0.5–60 kDa)

This class encompasses larger peptides and proteins, including β2-microglobulin (β2M) and α1-macroglobulin, which cannot be adequately cleared by conventional dialysis. Their removal typically requires high-flux dialyzers equipped with larger pore membranes [139]. Babb et al. described solutes within the range of 500 Da to 5000 Da as middle-molecular-weight solutes (MMS), which have been associated with increased mortality and the development of long-term complications such as dialysis-related amyloidosis, cardiovascular disease, and microinflammation in the chronic hemodialysis population [140,141].

iii. Protein-Bound Uremic Toxins (PBUTs)

The third category includes low-molecular-weight toxins that exhibit strong binding affinity to plasma proteins, predominantly albumin [65,73,142]. Due to this high binding capacity, PBUTs are poorly cleared by standard dialysis techniques and present a significant clinical challenge in the management of uremia [143]. Their persistent accumulation is implicated in a range of pathological processes, necessitating the development of targeted strategies to enhance their clearance.

### 5.2. Urea

The elimination of urea remains a major challenge in the context of dialysate regeneration [55,144]. In conventional single-pass hemodialysis, urea is efficiently removed from the blood into the dialysate compartment through diffusion [55,144]. However, this process becomes significantly more complex in closed-loop dialysate systems, such as those employed in Wearable Artificial Kidneys (WAKs) [55,106]. It is important to note that the kinetics of urea removal during dialysis do not accurately reflect the clearance efficiency of all uremic solutes [139]. Hasanzadeh et al. emphasized that small uremic toxins, particularly urea, represent the most substantial barrier to effective dialysate regeneration [104].

### 5.3. Other Uremic Toxins

Creatinine serves as a key uremic toxin whose accumulation in the blood instigates a cascade of adverse effects, exacerbating renal decline [145]. Uremic toxicity is linked to endothelial and immune dysfunctions, including inflammation and activation of innate immune responses, such as Toll-like receptors and pro-inflammatory cytokines [146,147]. Standard hemodialysis membranes are suboptimal for the complete removal of uremic toxins, particularly larger solutes [125,148]. Certain uremic toxins are resistant to absolute clearance by conventional dialysis techniques [73,123], leading to their accumulation in the body and contributing to various uremic disorders [73]. For example, clearance rates for p-cresol and creatinine are nearly 30% and 66%, respectively [71,149]. Such protein-bound uremic toxins (PBUTs) are involved in the increased cardiovascular risk in CKD patients [65,150].

PBUTs are low-molecular-weight, hydrophobic toxins with specific structural elements leading to binding to human serum albumin (HSA) via hydrophobic, electrostatic, and Van der Waals interactions [151,152]. In spite of their well-documented adverse effects on cardiovascular health [153,154], kidney failure [155] and mortality, their renal clearance mechanisms remain to be fully elucidated. Traditional dialysis is inefficient at removing PBUTs due to their high protein-binding affinity [156,157]. As a result, additional therapeutic strategies are necessary for adequate PBUT removal [158]. Prime examples of such PBUTs include indoxyl sulfate (IS) and p-cresyl sulfate (PCS), both indicating protein-binding fractions exceeding 90% [73]. Multiple studies have shown the harmful effects of IS on renal and vascular diseases [157,159].

### 5.4. Bilirubin

Bilirubin, a low- to middle-molecular-weight hydrophobic bile pigment, can contribute to multi-organ dysfunction when significantly elevated; neurologic injury (kernicterus) is a particular risk in neonates, and severe hyperbilirubinemia has been linked to renal impairment [4,5,122]. Adsorbents for bilirubin play a critical role in the treatment of hyperbilirubinemia [105]. In plasma, bilirubin is transported tightly bound to human serum albumin and is conjugated in hepatocytes with glucuronic acid before biliary excretion. In extracorporeal management of hyperbilirubinemia, bilirubin-selective sorbents and activated-carbon hemoperfusion have been investigated/used to enhance removal [160,161,162]. From the viewpoint of material science, various composite and matrix configurations have been developed for bilirubin adsorption. For instance, Chitosan/GO (CS/GO) aerogel microspheres have demonstrated strong stability and reusability in bilirubin adsorption [163,164].

Freeze-dried macro porous reduced GO aerogels reinforced with chitosan have been shown to demonstrate improved bilirubin adsorption capacities [164]. Novel adsorbents such as Chitin/Graphene Oxide (Ch/GO) composite aerogel beads have been developed for efficient and secure bilirubin removal [165]. Wu et al. engineered nanoporous CS/GO composite microspheres with superior bilirubin adsorption features [163].

SiO_2_-loaded graphene composite beads show not only mechanical strength but also outstanding blood compatibility, making them valuable candidates for bilirubin adsorption in hemoperfusion [166]. Functional CS/GO composite aerogel microspheres, synthesized via CO_2_ supercritical drying, have also shown remarkable efficacy in bilirubin removal [163].

In patients with CKD, the kidneys struggle to efficiently remove metabolic by-products like urea, creatinine, and bilirubin, leading to systemic complications such as cardiovascular disease [167,168]. In CKDs, toxins are classified according to their molecular weight and protein-binding characteristics, which influence their removal efficiency during dialysis [137]. Conventional single-pass hemodialysis is generally effective for urea removal, but other methods face challenges, particularly in closed-loop systems [105]. Uremic toxicity, often exacerbated by creatinine accumulation, impacts endothelial and immune function, requiring advanced dialytic approaches for comprehensive toxin removal [71,169]. The accumulation of bilirubin presents additional challenges; however, innovative adsorbents, especially those utilizing chitosan/GO composites, demonstrate promising effectiveness in its removal.

Graphene Oxide has excellent hydrophilicity and increased membrane permeability when incorporated into dialysis membranes; its high specific surface area and tunable pore structure improve selective toxin adsorption and solute clearance (e.g., creatinine); it also exhibits antioxidant properties that may reduce oxidative stress during dialysis [133]. Additionally, GO-enhanced membranes show improved mechanical strength and biocompatibility, reducing clotting and inflammation [170,171]. However, activated carbon is a strong adsorbent due to its high porosity and surface area for binding diverse toxins, but conventional AC membranes are thicker and less permeable than graphene-based membranes, limiting clearance rates in dialysis. AC has good capacity for adsorption of organic toxins but less so for integration into high-performance hemodialysis membranes [172,173]. Activated Carbon is often blended with reduced graphene oxide to combine their benefits, achieving improved adsorption and electrical properties; however, standalone activated carbon membranes are bulkier and less selectively permeable compared to GO-based membranes [173].

## 6. Mechanism of Adsorption by Carbon-Based Materials

Recent research has made substantial progress in elucidating the adsorption mechanisms of key uremic toxins through the application of carbon-based materials, which represent promising candidates for enhancing dialysis and hemoperfusion technologies [69,71,78,174,175,176,177]. Yuan et al. [48] employed computational simulations to design three affinity ligands based on the molecular structure of the uremia-associated peptide Asp–Phe–Leu–Ala–Glu (DE5) [48]. These ligands exhibited high selectivity and strong adsorption potential, driven by a synergistic interplay of coordination and hydrophobic interactions, thus providing critical insights into molecular-level adsorption behavior [48].

Among protein-bound uremic toxins, phenylacetic acid, indoxyl sulfate, and p-cresyl sulfate pose significant challenges for clearance due to their aromatic, hydrophobic nature and dissociable anionic groups, which require adsorbent surfaces capable of facilitating π–π interactions and electrostatic binding [150]. These characteristics require the design of materials with tuned surface functionalities.

In contrast, small water-soluble toxins such as urea, creatinine, and uric acid, which are present at higher concentrations in the bloodstream, primarily adsorb onto activated carbon (AC) through hydrogen bonding and Van der Waals forces [69,71,78]. The co-adsorption of these molecules onto porous AC results in multilayer physical adsorption, a process described effectively by pseudo-second-order kinetics and best fit by Freundlich and Halsey isotherm models [71]. These models confirm the dominance of physisorption, consistent with low activation energies [69,71,178,179].

Kinetic and thermodynamic modeling further supports these findings. For example, urea adsorption is predominantly governed by dipole–dipole interactions, including interactions with surface oxygen groups on AC [69,71]. In comparison, creatinine exhibits stronger adsorption, attributable to its lower polarity and enhanced affinity for the hydrophobic AC surface [69,71,180]. Uric acid, characterized by its high hydrophobicity and limited aqueous stability, engages primarily through hydrophobic interactions, resulting in a higher equilibrium adsorption capacity relative to urea and creatinine [71,181].

The adsorption behavior of bilirubin adds further complexity, as it circulates in plasma bound to albumin [23]. Effective bilirubin removal requires materials capable of displacing it from albumin complexes, such as chitosan/graphene oxide aerogels and three-dimensional porous graphene structures, which exploit π–π stacking and electrostatic interactions [23,71,163,178,182]. Experimental data show that bilirubin adsorption is influenced by pH, ionic strength, and temperature, with elevated temperatures enhancing adsorption due to conformational transitions in the bilirubin molecule [163,178].

Table 5 and Table 6 in the manuscript systematically present the primary and secondary interactions, as well as the activation energies associated with various uremic toxins. Notably, while urea and creatinine adsorption processes are consistent with physisorption, bilirubin exhibits both physical and chemical adsorption features, as evidenced by its activation energy (17.73 kJ/mol) [70,71].

**Figure 2:**
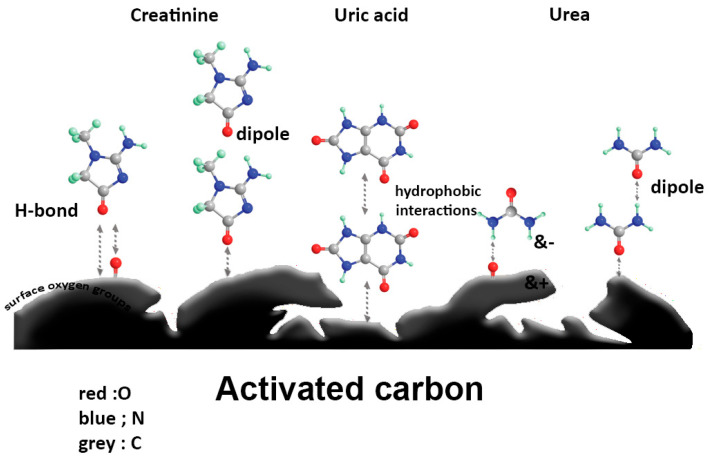
Schematic of adsorption mechanisms between representative uremic toxins and carbon-based adsorbents.

Figure 2 offers a visual synthesis of the adsorption mechanisms between uremic toxins and carbon-based adsorbents, highlighting surface functional group interactions (e.g., π–π stacking, electrostatic forces, dipole–dipole interactions, and hydrogen bonding), multilayer adsorption dynamics, pore size distribution, and the sequential saturation of adsorption sites.

Optimizing carbon-based adsorbents for uremic toxin removal hinges on a deep understanding of the physicochemical interactions governing adsorption [71]. Materials such as graphene, carbon nanotubes, and functionalized aerogels provide distinct advantages, including high surface area, tunability, and chemical specificity [48,69,71,163,178]. These attributes are pivotal for the development of next-generation adsorptive devices, aiming to improve patient outcomes in end-stage renal disease through enhanced extracorporeal blood purification.

## 7. Therapeutic Applications of Carbon-Based Materials for Blood Purification

### 7.1. Activated Carbon: Hemocompatible Adsorbents for Renal Support

Robust mechanical integrity and well-defined geometry are prerequisites for effective adsorbents [192]. It is widely hypothesized that spherical morphology may be the ideal shape for adsorbents, spherical particles offer low pressure drop and uniform flow distribution while preserving the material’s internal surface area accessible via porosity [34]. The produced AC spheres exhibit exceptional mechanical strength, elevated mesoporous volume, and efficient adsorption of medium-molecular-weight toxins [193]. A notable example of a medical-grade activated charcoal product utilized in clinical practice are the Baxter Charcoal Hemoperfusion Cartridge (Adsorba 300C-101223, developed by Baxter Healthcare, Deerfield, IL, USA) and Depuro D2000 Adsorption Cartridge (contains activated uncoated coconut shell (carbon granules) charcoal) [194,195]. The Baxter Charcoal Hemoperfusion Cartridge is designed specifically for hemoperfusion procedures and incorporates high-purity activated carbon with optimized pore structure to effectively adsorb endogenous and exogenous toxins from the bloodstream during extracorporeal circulation [50,194].

#### 7.1.1. Integration of Activated Carbon in Hemodialysis Membranes and Dialysate

Hemoperfusion (HP) often achieves higher clearances than conventional HD for certain poorly dialyzable toxins (e.g., paraquat), including many that are highly protein-bound [121,190]. Conventional HD struggles to remove persistent blood toxins (PBTUs), which are largely protein-bound [54,191]. Emerging technologies such as Hollow Mesoporous Carbon Spheres (HMCSs), Mixed Matrix Membrane Adsorbers (MMMAs), and dual-layer membranes aim to integrate adsorption and diffusion for improved toxin clearance [20,121]. Activated carbon (AC) has been embedded in membranes or introduced into dialysate streams, resulting in enhanced removal rates of solutes like indoxyl sulfate and p-cresol sulfate by over 70% [74,192]. These innovations contribute to closed-loop HD designs and wearable artificial kidney systems, offering promise for more efficient and sustainable dialysis treatments [121].

#### 7.1.2. Hemoperfusion with Modified Activated Carbon Adsorbents

Various commercial hemoperfusion systems have employed activated carbon (AC) and other adsorbents for the removal of medium to large molecular weight toxins, such as cytokines and LDL [13,191,196]. Modified mesoporous AC materials have demonstrated biocompatibility and effective cytokine removal, showing promise for sepsis and toxin management [197,198,199]. Despite their efficacy, direct contact with AC can lead to blood cell loss, necessitating further material optimization [33,198,200]. Advances such as encapsulation with PHEMA, CNT-based carbon spheres, and surface modifications (e.g., cellulose acetate coatings, hydrophilic grafting) have improved the biocompatibility and selectivity of these adsorbents [199,201]. Clinically, hemoperfusion remains a valuable tool in treating acute poisonings and reducing persistent blood toxins [199,201].

#### 7.1.3. Oral Carbon Adsorbents for Toxin Removal in CKD

AST-120, approved in various Asian countries in 1991, offers a strategy for delaying the initiation of dialysis in progressive CKD patients [119,202,203]. Comprising water-insoluble, spherical porous carbon particles with diameters ranging from 0.2–0.4 mm, AST-120 (also known as KREMEZIN) acts as an oral carbon adsorbent [119]. It captures uremic toxins and their precursors in the gastrointestinal tract, promoting their excretion through feces. Toxins such as indoxyl sulfate and p-cresyl sulfate, prevalent in the blood of CKD patients, are linked to CKD progression and cardiovascular disease [119]. DW-7202, a novel oral adsorbent consisting of black, spherical carbon particles (0.2–0.5 mm diameter) derived from a furan resin-based formulation, indicates high adsorptive selectivity for uremic toxins [204]. Comparative studies have evaluated patient preference, adherence, treatment efficacy, and safety between DW-7202 and AST-120 in pre-dialysis CKD populations [204]. The results suggest DW-7202 as a viable alternative for patients who have poor tolerance or adherence to granular adsorbents such as AST-120 [204].

### 7.2. Graphene-Based Nanocomposites for Blood Purification

#### 7.2.1. Functional Properties of Graphene Materials in Blood Purification

Graphene’s inherent hydrophobicity enables effective adsorption of PBUTs. However, surface chemistry variations influence biocompatibility [205]. Studies emphasize optimizing hemocompatibility for clinical use [113,206]. Graphene composites have shown promise in adsorbing bilirubin, creatinine, and urea, especially when functionalized with chitosan or immobilized enzymes.

#### 7.2.2. Hemodialysis Systems Using Graphene-Based Materials

Graphene oxide (GO) and reduced GO (rGO) integrated into dialysis membranes have demonstrated enhanced removal of solutes such as urea and indoxyl sulfate [207,208]. Urease-immobilized GO systems have achieved >75% urea clearance in in vitro simulations [207]. Variables like dialysate flow rate and GO concentration significantly influence efficacy [208]. Thin-film nanofibrous composites and mixed-matrix membranes represent promising configurations for next-generation HD devices [205,207].

#### 7.2.3. Hemoperfusion Using Graphene Composites

In HP applications, GO-based composites like CS/GO aerogel microspheres, silica-loaded GO beads, and chitin/GO hybrids show high bilirubin adsorption with minimal hemolysis [163]. These structures demonstrate mechanical durability, reusability, and excellent blood compatibility [163]. They also reduce infiltration resistance and clotting risk, making them superior candidates for advanced hemoperfusion columns.

#### 7.2.4. Commercial Integration of Graphene in Dialysis Devices

Commercial hollow-fiber dialyzers were effective for rapid purification of GO [209]. Increased dialysate flow rates significantly reduced purification time due to higher volumetric replacement of the dialysate [209].

## 8. Conclusions and Future Prospects

It is well known that CKD patients fail to filter blood waste due to progressive and irreversible damage to the kidney’s filtering units over time, resulting in the accumulation of uremic toxins, fluid overload, electrolyte imbalances, metabolic acidosis, and chronic anemia. Moreover, these patients face persistent inflammation and oxidative stress, due to limited abilities of conventional dialysis to remove middle toxin molecules and inflammatory mediators. As a result, they are prone to cardiovascular risks, reduced quality of life, and higher mortality rates.

Carbon-based materials have demonstrated great potential in hemodialysis and hemoperfusion owing to their remarkable surface area, chemical stability, and ease of modification. They can be integrated into dialysis systems, in order to enhance toxin clearance, reduce oxidative stress, and improve biocompatibility. Their multifunctional properties allow the development of next-generation blood purification equipment for renal patients. Their natural hydrophobicity makes them highly effective for absorbing hydrophobic toxins, while their mechanical and chemical properties enhance the efficiency and selectivity of toxin removal processes. Their major mechanism of actions to collect toxins are via physical adsorption (Van der Waals Forces), π–π Interactions, hydrophobic interactions, electrostatic and hydrogen bonding interactions, redox activity and reactive oxygen species (ROS) scavenging. Research has shown that graphene-based nanomaterials are effective in removing a wide range of toxins, such as bilirubin and persistent blood toxins. Their outstanding compatibility with blood also helps reduce coagulation and infiltration resistance. ACs and CNTs are both promising materials for blood filtration, but they differ significantly in performance and application. AC is widely used due to its high surface area, broad-spectrum adsorption, low cost, and clinical safety; however, it lacks selectivity and may adsorb essential molecules. CNTs, on the other hand, offer higher surface area and tunable selectivity through functionalization, making them suitable for targeted toxin removal, but concerns about cytotoxicity and higher production costs limit their blood purification use. Compared to other carbon-based materials, ACs remain the most practical for blood purification applications, while GO shows emerging potential for membrane development with improved selectivity. Overall, each material has unique filtration properties, with ACs being favored for general detoxification applications.

Future research on graphene-based materials for blood purification must tackle a number of challenges to completely realize their potential. Optimizing hemocompatibility is crucial to ensure safe interactions with blood components, while surface modification and functionalization can enhance toxin selectivity and efficiency. Scalability and cost-effective manufacturing processes are needed to produce high-quality graphene materials at a commercial scale. Guaranteeing the long-term stability and reusability of these materials during repeated use is crucial for their practical applications. In-depth toxicological research is necessary to evaluate the potential long-term impacts of exposure to graphene. Combining these materials with current hemodialysis and hemoperfusion systems, along with fostering interdisciplinary partnerships and conducting thorough clinical trials, will facilitate the transition from laboratory discoveries to clinical use. Furthermore, creating new graphene-based composites with customized properties targeting specific toxins and clinical requirements will propel the field ahead, providing enhanced treatment alternatives for patients needing toxin removal.

## Figures and Tables

**Table 1:** Advantages and Limitations of Conventional Therapeutic Approaches in Renal Disease Management.

Various Blood Perfusion Treatment	Description	Substances Removed	Advantages	Limitations/Disadvantages	References
**Hemodialysis**	Purifying blood indirectly using a device that filters out waste products.	- Small Molecular Weight - Water-soluble substances - Low removal of protein-bound solutes	- Widely available, well-established treatment that can be performed in hospital or at home (flexible). - Effective at removing small, water-soluble uremic toxins and excess fluid.	- Limited in removing larger protein-bound toxins - Associated with high morbidity and mortality rates. - Requires frequent sessions, impacting quality of life. - Time-consuming (3–5 h per session, multiple times a week). - May not replace all kidney functions Potential for adverse reactions and complications.	[13,55,56,57,58]
**Hemoperfusion**	Using direct carbon contact with blood to remove toxins via an extracorporeal circuit.	- Middle molecular weight & protein-bound uremic toxins - Substances adsorbed on activated carbon	- Enhances removal of medium to large uremic toxins, improving patient outcomes. - When combined with hemodialysis (HP + HD), adds adsorption to diffusive/convective clearance for broader toxin coverage. - Reduces complications associated with long-term dialysis.	- Limited availability and higher costs. - Short-term procedure, often requiring repeated treatments.	[17,57,59,60,61,62,63,64]
**Oral treatment**	Removing toxins from the digestive system	Hepatically metabolized substances	- Non-invasive and convenient for patients. - Can slow disease progression and address complications like hyperkalemia or anemia with specific drugs.	- Effectiveness depends on the stage of kidney disease. - Limited to managing symptoms and slowing progression, not a replacement for kidney function.	[13,52,53,57]

**Table 2:** Advantages and Limitations of Diverse Carbon-Based Materials in Blood Perfusion Therapy.

Various Carbon	Advantages	Limitations/Disadvantages	References
**Activated Carbon**	- Good removal efficiency - High specific area - low cost - Surface activity - Can be easily modified	- Limited hemocompatibility - Difficult to remove after dosing - Temperature sensitive	[48,68,69,71,78]
**Graphene Oxide (GO)**	- Low cost - Easy to modify surface activity - Water dispensability - Polar functionalization	- Surface random functionalization - Limited hemocompatibility - Lower electrical and thermal conductivity - Poor control on post-preparation functionalization	[23,79,80,81,82]

**Table 3:** Physical characteristics and applications of different types of Activated Carbon.

Activated Carbon Type	Physical Characteristics	Typical Applications	References
**Powdered-activated carbon** **(PAC)**	Particle size <1.0 mm, typically between 0.15 and 0.25 mm	Rapid adsorption due to high surface area	[56,91]
**Granular Activated Carbon (GAC)**	Large particle size than PAC; lower external surface	Adsorption in liquid and vapor phases	[56,92]
**Extruded activated carbon (EAC)**	cylindrical shape, sizes ranging from 0.8 to 45 mm	Primarily used in gas-stage applications	[56,91,92]
**Bead activated carbon (BAC)**	spherical shape; smaller size than EAC	Fluidized bed applications	[92]
**Impregnated coated carbon** **(ICC)**	permeable structure	Air pollution control	[56,91]
**Polymer coated carbon (PCC)**	Biocompatible polymer coating, smooth and porous without blocking pores	Medical applications, e.g., hemoperfusion	[56,92]

**Table 4:** Key points about activating agents and chemical activation for producing activated carbon.

Definition of Activating Agents	Compounds Included in the Precursor Formulation for Producing Activated Carbon (AC)	References
**Common Activating Agents**	KOH, ZnCl_2_, H_3_PO_4_, NaOH, Ca (OH)_2_, K_2_CO_3_, FeCl_3_.	[56,92,108,109]
**Functions of Activating Agents**	- Promote pore formation in AC. - Serve as dehydrating agents to capture moisture. - Stabilize the final product by facilitating intermolecular force arrangement. - Elevate activation temperatures and micropore volumes.	[56,92,108,109]
**Chemical Activation Method**	Single-step preparation involving soaking the carbonaceous precursor in a dehydrating agent followed by activation at high temperatures under an inert atmosphere.	[56,92,108,109]
**Alternative Activating Agents**	Alkali metal carbonates (e.g., K_2_CO_3_) and alkali earth metal salts (e.g., FeCl_3_, ZnCl_2_) can replace alkali metal hydroxides due to their corrosive nature.	[56,92,108,109]
**Limitations of Certain Agents**	- ZnCl_2_: Not suitable for pharmaceutical and food industries due to contamination risks. - H_2_SO_4_ and H_3_PO_4_: Toxicity and high costs limit their use.	[56,92,108,109]
**Effect of Alkali Metal Carbonates**	Different alkali metal carbonates (Li_2_CO_3_, Na_2_CO_3_, K_2_CO_3_, Rb_2_CO_3_, Cs_2_CO_3_) show a direct relationship between the agent used and the surface area of resulting AC.	[56,92,108,109]
**Advantages of Chemical Activation**	- Lower activation temperature. - Single-step operation. - Shorter drying treatment. - Higher carbon content.	[56,92,108,109]
**Disadvantages of Chemical Activation**	- High cost of activating agents. - Requires additional washing steps.	[56,92,108,109]
**Physical Characteristics of Activated Carbon**	- Smooth, non-homogenous granules. - Average size: 0.5–1.0 mm. - Large surface area.	[56,92,108,109]
**Phenol Formaldehyde Activated Carbon (PFAC)**	- Coarse-mesh charcoal. - Cinder content < 0.05%. - Synthesized by carbonization of phenol formaldehyde resin.	[56,92,108,109]

**Table 5:** Challenges and Solutions for toxins in CKD and dialysis.

Topics	Details	Challenges & Solutions	References
**Toxins in CKD**	- Healthy kidneys remove metabolic by-products. - Toxins categorized into small, middle, and protein-bound uremic toxins.	- Accumulation of toxins in CKD leads to systemic complications.	[73,179,183,184,185,186,187]
**Toxins in Dialysis** **Urea**	- Easily removed in conventional single-pass hemodialysis.	- Difficult to remove in closed-loop WAK systems. - Not representative of all uremic solutes.	[106,136,138,174,188]
**Uremic**	- Creatinine is a major uremic toxin. - Leads to endothelial and immune dysfunction.	- Conventional dialysis inadequate for all uremic toxins.	[1,3,10,17,47,49,125]
**Bilirubin**	- Excessive bilirubin leads to multiorgan dysfunction.	- Various adsorbents developed for removal. - Chitosan/Graphene oxide aerogels show promise.	[67,163,165,189,190,191]

**Table 6:** Interactions, activation energy, adsorption, and equilibrium of blood toxins with activated carbon.

Molecule	Primary Interaction	Additional Interactions	Activation Energy (kJ/mol)	Multilayer Adsorption	Time to Reach Equilibrium	References
**Urea**	Dipole–dipole	H-bonding, surface oxygen groups	from –50.6 to –70.1	Yes	Not specified	[69,70,71]
**Creatinine**	Van der Waals	H-bonding, dipole-induced dipole, surface oxygen groups	−4.9	Yes	Not specified	[70,71]
**Bilirubin**	π-π, electrostatic	H-bonding, hydrophobic	17.73	Not specified	<120 min	[13,69,162]
**Uric Acid**	Hydrophobic	Van der Waals	14.2	Yes	Not specified	[70,181]

## Abbreviations

The following abbreviations are used in this manuscript:

ACs	Activated Carbons
CKD	Chronic Kidney Disease
PCS	P-Cresyl Sulfate
OSCA	Oral Spherical Carbon Adsorbent
IL-6 & IL-8	Serum and Urine Interleukin-6 and Interleukin-8 Levels
ESRD	End Stage Renal Disease
SWCNTs	Single Wall Carbon Nanotubes
MWCNTs	Multi Wall Carbon Nanotubes
HD	Hemodialysis
REDY	Regenerative/Recirculating Dialysate
PAC	Powdered Activated Carbon
GAC	Granular Activated Carbon
EAC	Extruded Activated Carbon
BAC	Bead Activated Carbon
ICC	Impregnated Coated Carbon
PCC	Polymer Coated Carbon
PFAC	Phenol Formaldehyde Activated Carbon
GO	Graphene Oxide
EUTox	Uremic Toxins
PBUTs	Protein-Bound Uremic Toxins
WAK	Wearable Artificial Kidneys
HSA	Human Serum Albumin
CS/GO	Chitosan/Graphene Oxide
Ch/GO	Chitin/Graphene Oxide
UF	Ultrafiltration
ΔG	Gibbs Free Energy Change value
ΔH	Enthalpy Change value
R^2^	Correlation Coefficient
3D-pGR	Three-Dimensional Porous Graphene
BSA	Bovine Serum Albumin
HP	Hemoperfusion
PQ	paraquat
HMCSs	Hollow Mesoporous Carbon Spheres
MMMAs	Mixed Matrix Membrane Adsorbers
PAH	Para-Amino Hippuric Acid
MMM	Mixed-Matrix Membrane
LDL	Low-Density Lipoprotein
DHP	Direct Hemoperfusion
PHEMA	Polyhydroxy ethyl Methacrylate
CNTs/P-ACSs	Carbon Nanotubes/Phenolic-Resin-Derived Activated Carbon Spheres
P-ACSs	Phenolic-Derived Activated Carbon Spheres
MDAC	Multiple-dose Activated Charcoal
CH	Charcoal Hemoperfusion
ACAC	Albumin-Cellulose nitrate-coated Activated Charcoal
TNFC	Thin-film Nanofibrous Composite
rGO	reduced GO
TRGO	Thermally Reduced Graphene Oxide
UPAS-MGO	Urease-Immobilized Magnetic Graphene Oxide
